# Major inducing factors of hypertensive complications and the interventions required to reduce their prevalence: an epidemiological study of hypertension in a rural population in China

**DOI:** 10.1186/1471-2458-11-301

**Published:** 2011-05-11

**Authors:** Min Zhang, Yong Meng, Yongli Yang, Yancai Liu, Caiqin Dong, Jianming Xiao, Ling Zhao, Fang Li

**Affiliations:** 1 Department of Cardiology, First Affiliated Hospital of Kunming Medical University, Kunming, Yunnan 650032, P.R. China; 2 Department of Cardiology, Second Affiliated Hospital of Kunming Medical University, Kunming, Yunnan 650101, P.R. China; 3 First Hospital of Binchuan County, Yunnan 671600, P.R. China; 4 Emergency Department, Second Affiliated Hospital of Kunming Medical University, Kunming, Yunnan 650101, P.R. China

**Keywords:** hypertension, complications, Chinese rural areas, the New Rural Cooperative Medical Scheme

## Abstract

**Background:**

The complications of hypertension cause severe health problems in rural areas in China. We (i) screened the major factors inducing hypertensive complications and provided intervention measures; and (ii) verified the efficacy of the New Rural Cooperative Medical Scheme (NRCMS; a medical insurance scheme for rural residents) for hypertension management.

**Methods:**

A survey was conducted in the villages of Yunnan (an underdeveloped province in southwest China). The NRCMS was initiated there in 2005. Data were collected through questionnaires, physical examination, electrocardiography, as well as blood and urine tests. To detect factors inducing hypertension complications, a generalized estimating equations model was developed. Multivariable logistic regression was used to analyze influencing factors for hypertension control.

**Results:**

Poor management of hypertension was observed in women. Being female, old, poorly educated, a smoker, ignorant of the dangerousness of hypertension, and having uncontrolled hypertension made patients more prone to hypertension complications. Combination therapy with ≥2 drugs helped control hypertension, but most rural patients disliked multidrug therapy because they considered it to be expensive and inconvenient. The NRCMS contributed little to reduce the prevalence of complications and improve control of hypertension.

**Conclusions:**

The present study suggested that the NRCMS needs to be reformed to concentrate on early intervention in hypertension and to concentrate on women. To increase hypertension control in rural areas in China, compound products containing effective and inexpensive drugs (and not multidrug therapy) are needed.

## Background

Hypertension is one of the leading causes of cardiovascular disease and premature mortality in the world [1]. Uncontrolled hypertension results in various complications (e.g., coronary heart disease, stroke, congestive heart failure, renal insufficiency, and peripheral vascular disease [2]), which are the major causes of morbidity and mortality. In China, hypertension is a common health problem with a rising prevalence. From 1960 to 2002, the number of hypertensive patients among Chinese adults rose from 30 million to 129 million [3,4]. However, the awareness, treatment, and control of hypertension are relatively poor. Among hypertensive patients in China, 44.7% are aware that they have high blood pressure, 28.2% are taking antihypertensive medications, and 8.1% achieve control of blood pressure [3]. The rates of awareness, treatment, and control are even lower in rural areas in China, although the prevalence of hypertension in rural areas is similar to that in urban areas [5]. China has 744.71 million people involved in agriculture, which is 57.01% of the total population of China [6]. The largest number of hypertensive patients is in rural areas. Numerous patients and poor management of hypertension in the countryside bring about serious complications which have become a heavy burden on the health system in China. Strengthening medical monitoring in rural areas and taking steps to reduce complications due to uncontrolled hypertension should be priorities for Chinese health authorities.

For rural residents not employed in formal employment, the Chinese government has been rolling out a medical insurance scheme called the "New Rural Cooperative Medical Scheme" (NRCMS) since 2003. It is a voluntary health insurance program funded by enrollee contributions and by subsidies from central and local governments. Households purchase health insurance for modest premiums of 10-20 Yuan RMB (~$1.50-3.00) per person per annum; local and central governments each contribute 20-40 Yuan RMB per enrolled individual [7]. The NRCMS is administered at the county level, so coverage has varied across regions of China and over time. County administrators define benefits packages on the basis of local needs and resources [8]. By 2005, the NRCMS in all pilot counties covered inpatient care; however, only one-quarter included outpatient expenses on a pooling basis. The bulk of reimbursement by the NRCMS was for inpatient expenses, even in counties that covered outpatient expenses [7]. To supplement the NRCMS, Medical Financial Assistance (MFA) was established in 2003. MFA is supposed to provide assistance mainly for the designated poor (who are identified by local governments according to the national extreme and relative poverty lines) to pay the NRCMS premium and part of the NRCMS non-reimbursable medical expenses [9]. Other medical insurance programs (e.g., private insurance) are rarely purchased by rural residents. The NRCMS is the largest health insurance program in Chinese rural areas. By 2010, the average coverage of the NRCMS throughout China has been ≥95%. This program is expected to reduce the financial burden on rural residents and improve their health status, but its effectiveness needs to be confirmed.

The aim of the present study was to screen the major factors inducing hypertensive complications among rural residents. Also, the effectiveness of the NRCMS for increasing control of hypertension and decreasing the prevalence of complications was evaluated to provide intervention measures to improve hypertension control and prevent complications in patients living in rural areas. The study was done in the mountainous areas of Yunnan province (an under-developed area in southwest China).

## Methods

The study protocol was approved by the Ethics Committee of Kunming Medical University (Yunnan, China). Each participant provided written informed consent.

### Study subjects

We identified four mountain villages in Binchuan County (an agricultural county with a low economic level in the west of Yunnan province) as the study sites. It joined the NRCMS in 2005, but was not the pilot county of MFA at the time of the survey. An investigation was made from February to April in 2007 (a short period before spring ploughing to ensure the maximum number of young adults stayed in their native villages instead of going out to work). There were 2,022 households, with 7,665 people in these villages. All villagers were peasants, and 71.2% of them were covered by the NRCMS. The household survey was conducted for all villagers aged ≥25 years.

### Measurement of blood pressure (BP)

The BP of participants was taken in their homes by trained technicians. BP was measured in the right arm supported at the heart level with a standardized mercury sphygmomanometer after a rest of ≥5 min. BP was measured twice with 30 s of rest between each measurement. The mean of two readings was used for analyses. Hypertension was defined as systolic blood pressure (SBP) ≥140 mmHg, and/or diastolic blood pressure (DBP) ≥90 mmHg, and/or reported treatment with antihypertensive agents in the past 2 weeks. The prevalence of awareness was defined as the percentage of hypertensive subjects reporting a prior diagnosis of hypertension. The prevalence of treatment was defined as the percentage of hypertensive patients with a history of therapy using antihypertensive drugs. The prevalence of control was defined as the percentage of hypertensive patients with BP controlled <140/90 mmHg.

### Collection of demographic and laboratory data

Demographic data were obtained from in-house surveys. Research staff administered a questionnaire to collect personal data (age, sex, height, weight, medical history, number of years of schooling, smoking status, alcohol consumption, household income, disposable income), as well as finding out if the participant had joined the NRCMS or other insurance schemes. For those considered to have hypertension, questions with regard to the awareness of high BP, knowledge of the dangerousness of hypertension, and treatment status were asked. Questions about medicine names, treatment cost, and the maximum acceptable cost of treatment per month were inquired among patients who received antihypertensive treatment.

Once subjects were defined as having hypertension, they were invited to village clinics to undergo electrocardiography (ECG) and to donate samples of blood and urine. A routine urine test was conducted and the level of creatinine in serum determined in the clinical laboratory of the First Hospital of Binchuan County (Yunnan, P. R. China) on the same day as sample collection. Electrocardiograms were delivered to the First Affiliated Hospital of Kunming Medical University (Kunming, P. R. China) to be analyzed by technicians unconnected to the study.

### Detecting the adverse outcomes of hypertension

For subjects with hypertension, the adverse outcomes of this disorder were detected. In the present study, outcomes were classified as "brain damage", "heart damage", "kidney damage", and "other". Hypertensive patients with histories of stroke and/or transient ischemic attack were placed in the "brain damage" category. Heart damage was ascertained as having any of the following: (1) ECG showing left-ventricular hypertrophy: Sokolow-Lyon index >38 mm or Cornell voltage product >2440 mm·ms [10]; (2) signs and symptoms of heart failure presented by excluding other causes through questions regarding medical history; and (3) patients with the typical chest pain associated with angina pectoris. Patients in whom routine urinalysis showed urine protein to be positive, and/or serum creatinine >1.5 mg/dL, were classified as having kidney damage. Other outcomes due to hypertension such as fundus bleeding and/or intermittent claudication were classified to the "other" category.

### Statistical analyses

Statistical analyses were undertaken using the Stata 10 statistical package. Continuous variables are presented as means and discrete variables as frequencies and percentages. The Student's *t*-test and one-way analysis of variance for continuous variables, and the chi-square test for categorical factors, were applied to compare across the groups. Next, to estimate the occurrence of hypertensive adverse consequences based on potential risk factors, a model was developed via generalized estimating equations (GEE). In this model, hypertension outcome was adopted as the dependent variable, and potential risk factors as independent variables. Hypertension outcomes were divided into four categories, and one hypertensive patient could experience one or more outcomes simultaneously. These were multiple response data in which different options in the multiple response data were not only interconnected, but also relatively independent. The data can be analyzed by building the model of GEE [11]. Subsequently, another model was developed to analyze the influence of factors on the control prevalence of hypertension among patients who received antihypertensive treatment. Multivariable stepwise logistic regression was used on this occasion. A two-sided P value of <0.05 was considered to be significant.

## Results

Among 7,665 villagers, 5,682 individuals were ≥25 years. Except those who were away from home or unwilling to be interviewed, 5,110 individuals were enrolled in this survey. Male subjects accounted for 51.9% (2652/5110). The prevalence of NRCMS coverage among all subjects was 70% (3577/5110). No participant purchased private medical insurance at the time of this survey.

### Prevalence, awareness, treatment, and control of hypertension

High BP or hypertensive history was detected in 1,340 subjects (714 males and 626 females). The prevalence of hypertension in all subjects was 26.2% (26.9% in males and 25.5% in females). There was no significant difference in the prevalence of hypertension between sexes (p = 0.237). Among all subjects defined as having hypertension, 436 subjects were aware of their high BP, 351 subjects were taking antihypertensive medicines, and 93 patients had their BP under control. The prevalence of awareness, treatment and control of hypertension was 32.4%, 26.2%, and 6.9% respectively.

### Data in different groups

The hypertensive group and normotensive group showed no significant difference in sex ratio, prevalence of NRCMS coverage, daily intake of alcohol, family income per month, and personal disposable income per month (p > 0.05). Subjects with hypertension were older and had a higher body mass index (BMI) than those without hypertension (p < 0.05). Smoking and lack of education were more common in the hypertensive group (p < 0.05). The data of male and female hypertensive patients are presented at Table 1. Female patients had a lower prevalence of medical-insurance coverage, higher occurrences of hypertensive outcomes, shorter duration in school, less disposable income, less treatment costs, and less maximum acceptable treatment cost. Family income was similar in the two groups. Compared with male patients, more female understood the danger of hypertension, but the prevalence of awareness, treatment and control of hypertension were lower in female patients. 

**Table 1:** Data of hypertensive patients in male and female groups

	Male	Female	*P *
Number	714	626	
Age (y)	65.5 ± 9.1	67.2 ± 8.7	0.0008
BMI (kg/m^2^)	29.7 ± 6.1	22.6 ± 3.3	<0.0001
Smokers (%)	95.0(678/714)	59.6(373/626)	<0.0001
Daily intake of alcohol (mL)	50.2 ± 6.5	28.8 ± 8.2	<0.0001
Number of years of schooling (y)	3.3 ± 2.7	1.3 ± 1.4	<0.0001
Insurance coverage (%)	73.1	66.3	0.007
Family income per month (Yuan RMB*)	684.6 ± 173.9	673.1 ± 149.5	0.1979
Personal disposable income per month (Yuan RMB*)	456.9 ± 188.9	292.2 ± 104.3	<0.0001
Monthly treatment cost (Yuan RMB*)	10.9 ± 9.6	5.9 ± 5.3	<0.0001
Maximum acceptable treatment cost per month (Yuan RMB*)	126.1 ± 83.8	65.1 ± 33.7	<0.0001
Understanding of the danger of hypertension (%)	12.9(92/714)	16.8(105/626)	0.045
Awareness (%)	36.0(257/714)	28.6(179/626)	0.004
Treatment (%)	30.1(215/714)	21.7(136/626)	<0.0001
Control (%)	9.1(65/714)	4.6(29/626)	0.001
Hypertension outcomes (%)	41.7(298/714)	48.4(303/626)	0.014

*Currency = Renminbi, currency unit = Yuan, US$1.00 = RMB 6.844

BMI: Body mass index.

### Antihypertensive agents used among hypertensive patients

Among patients receiving antihypertensive treatment, the commonest agents are the compound drugs listed below.

• Compound tablet of kendir leaves: each tablet contains guanethidine (1.3 mg), hydralazine (1.6 mg), hydrochlorothiazide (1.6 mg), and kendir leaves (218.5 mg).

• Compound tablet of pearl powder and *Chrysanthemi flos*: each tablet contains clonidine (0.03 mg), hydrochlorothiazide (5 mg), *Chrysanthemi flos *powder (10 mg), pearl powder (100 mg), and melin (20 mg).

• Compound antihypertensive tablet: each tablet contains reserpine (0.03125 mg), potassium chloride (30 mg), hydrochlorothiazide (3.125 mg), and hydralazine (3.125 mg).

• Compound tablet of dihydralazine sulfate and hydrochlorothiazide: each tablet contains dihydralazine (5 mg), clonidine (0.015 mg), and hydrochlorothiazide (5 mg).

**Figure 1:**
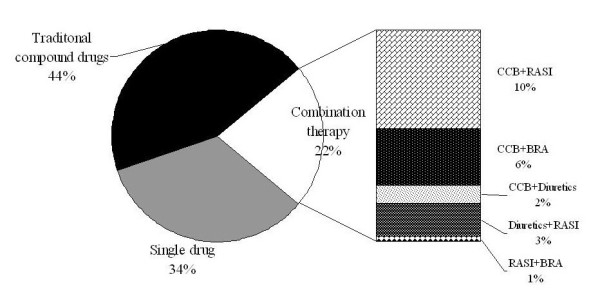
**Antihypertensive therapeutic scheme among hypertensive patients undergoing treatment**. CCB: calcium channel blockers. RASI: rennin-angiotensin system inhibitors. BRA: beta receptor antagonists.

Figure 1 details the antihypertensive therapeutic protocols. A total of 44.0% of patients took one type of the compound drugs described above. The remaining antihypertensive agents were calcium-channel blockers (35.3%), inhibitors of the renin-angiotensin system (17.2%), diuretics (14.4%), and β-receptor antagonists (8.0%). Patients administered compound products were no longer treated with other antihypertensive drugs. According to their therapeutic protocols, patients who received drug therapy were divided into three groups. These were the compound drugs group (taking one of compound products mentioned above, 44%), single drug group (taking other single drugs, 34%), and multidrug therapy group (treated with ≥2 drugs, 22%). Of these patients, 78% were treated with one single drug. Treatment with a single drug rather than multiple drugs was regarded as the most inexpensive and convenient by most subjects. The number of medications used was similar between males and females. The total treatment cost among patients receiving antihypertensive treatment was 3141 Yuan RMB per month, and the proportion of reimbursement by the NRCMS was 2.5%. Among single-drug, compound-drugs and multidrug therapy groups, the treatment cost per month showed a significant difference (5.85 *vs*. 13.49 *vs*. 15.35, respectively), but there was no significant difference between the compound drugs group and multidrug group (p = 0.118).

### GEE Model

Before developing the GEE model, a high correlation was found in three variables which reflected the economic status of subjects: family income per month, personal disposable income per month, and maximum acceptable treatment cost per month (r = 0.72-0.91, p < 0.01). To eliminate the interference of multi-variable collinearity, principal components analysis (PCA) was adopted. Three principal components were extracted based on PCA. The variance contribution of the first principal component had reached 86.5% (which was sufficient to mirror the information of the original three variables), so the first principal component was named "personal economic condition" to go to the next analysis. The expression was:

A GEE model was fitted (Table 2). Four constants were fitted into this model to reflect the natural logarithm of the odds ratio (OR) of the prevalence of four hypertension outcomes on the baseline. Adjusted by all variables, the prevalence of hypertensive outcomes was 0.69‰ (brain damage), 0.13‰ (heart damage), 0.26‰ (kidney damage), and 0.02‰ (other). The results of the GEE model showed that being female, old, smoking tobacco products, and having awareness of hypertension were the promoting factors to adverse outcomes of hypertension (p < 0.001, respectively). Conversely, a longer duration of schooling (p = 0.014), better personal economic condition (p < 0.001), understanding the danger posed by hypertension (p = 0.002), and well-controlled BP (p < 0.001) were inhibitory factors for hypertension outcomes. Having medical insurance (p = 0.925) and receiving hypertensive treatment (p = 0.782) did not contribute to the prevention of the adverse outcomes of hypertension.

**Table 2:** Results of generalized estimating equations

	Coefficient	Standard error	z	*P*
Cons_1	-7.27491	0.8533082	-8.53	0
Cons_2	-8.921791	0.8670381	-10.29	0
Cons_3	-8.253619	0.8607792	-9.59	0
Cons_4	-10.71109	0.8952318	-11.96	0
Female (*vs*. male)	1.389162	0.1959131	7.09	0
Age	0.0532048	0.0079643	6.68	0
BMI	-0.114449	0.009973	-1.15	0.251
Joining NRCMS (*vs*. not joining)	-0.009856	0.1041018	-0.09	0.925
Daily smoking (*vs*. not smoking)				
<10 cigarettes	0.6989892	0.1490481	4.69	0
10-20 cigarettes	1.880096	0.1982579	9.48	0
21-30 cigarettes	4.164752	0.2797552	14.89	0
31-40 cigarettes	5.027749	0.3877939	12.97	0
>40 cigarettes	5.423671	0.4484097	12.1	0
Daily alcohol intake (*vs*. not drinking)				
<50 mL	-0.144686	0.1352986	-1.07	0.285
50-100 mL	0.0492849	0.1462375	0.34	0.736
>100-150 mL	0.2496853	0.371617	0.67	0.502
>150-200 mL	0.9168573	0.4639914	1.98	0.048
>200 mL	0.1791324	0.870807	0.21	0.837
Number of years of schooling	-0.077254	0.0313048	-2.47	0.014
Financial capacity	-0.213526	0.0570027	-3.75	0
Awareness of hypertension	1.545984	0.1657462	9.33	0
Understanding the danger due to hypertension	-0.585789	0.1877989	-3.12	0.002
Treatment of hypertension	-0.052023	0.1910931	-0.27	0.785
Control of hypertension	-1.67305	0.3589172	-4.66	0

_1 Brain damage

_2 Heart damage

_3 Kidney damage

_4 Other

NRCMS: New Rural Cooperative Medical Scheme, BMI: body mass index

### Logistic regression model

The results of stepwise logistic analysis are shown in Table 3. Combination therapy with ≥2 drugs, understanding the danger due to hypertension, compound drugs therapy, and higher treatment cost were beneficial to hypertension control. Conversely, being female and smoking ≥20 cigarettes per day were not beneficial to hypertensive control. Joining the NRCMS and receiving antihypertensive treatment were excluded from this model.

**Table 3:** Results of logistic regression analysis

	Coefficient	Odds ratio	z	*P *
Constants	-7.952181		-6.32	0
Multidrug therapy	3.763052	43.07971	5.49	0
Understanding of the danger of hypertension	4.118705	61.47958	7.2	0
Female	-1.529124	0.2167254	-3.39	0.001
Traditional compound drugs therapy	1.404991	4.075489	2.65	0.008
Treatment cost	0.0824604	1.085956	2.63	0.009
More than 20 cigarettes per day	-2.182919	0.112712	-2.32	0.02

## Discussion

Hypertension is a common health problem in 'developing' countries [12]. We revealed an overall prevalence of hypertension of 26.2% in Yunnan province. The prevalence of hypertension was 26.9% in men and 25.5% in women. The general prevalence of awareness, treatment and control were lower than that observed in adults in the USA [13] and the mean level of Chinese adults [3]. That is, hypertension is a serious health problem in rural areas of China.

The World Bank in 2009 [14] reported that the number of people living in poverty in China in 2005 was 254 million, and that the poor were predominantly from rural areas. The prevalence of hypertension was similar in rural and urban areas, but treatment and control in rural areas was not good [5]; this was largely due to the cost of obtaining medical treatment [7]. The medical insurance program provided by the Chinese government is particularly needed by farmers.

The NRCMS focuses largely on the costs of inpatient care rather than the costs of basic services (including personal and communal preventive interventions); also, reimbursement of outpatient expenses can be difficult to obtain. A recent study [15] showed that the NRCMS could not protect against financial catastrophe and household impoverishment. For most chronic diseases such as hypertension, inpatient care is at a late stage of medical intervention, which is expensive. Early interventions (including preventive care and outpatient care) are more cost-effective than inpatient care. We showed that smoking, lacking knowledge of the dangerousness of hypertension, and having uncontrolled hypertension made hypertensive subjects prone to various complications. Most patients seldom realized their high BP status until complications occurred (which could explain why awareness of hypertension became a promotion factor to induce hypertensive complications). Education regarding smoking cessation, improving people's understanding of hypertension, and regular follow-up in clinics are cost-effective methods to increase hypertension control and decrease its complications. However, the costs of these methods are neglected by the NRCMS. This could be why the NRCMS does not help to improve hypertension management. Another study revealed [16] that adding outpatient reimbursement to the benefit package encouraged poor families to use healthcare services and thus reduced their potential need for hospitalization and its financial burden.

Prevalence of the awareness of hypertension and treatment in women is higher than that in men [3,5,13,17-20], and good control of BP in women more readily obtained. However, the present study showed poorer management of hypertension in women. Female patients had a lower degree of education as well as less medical-insurance coverage. Even if their family income was similar to that of men, women had less disposable income, treatment costs, and maximum acceptable treatment cost. This mirrored the second-class status that women have within the family and society in China. Should be the NRCMS change so that it helps rural women? Should a special fund be established for the healthcare of rural women? Could new medical reforms resolve these problems? Answers to all of these questions are needed.

Multiple studies have shown that good control of BP is vital to avoiding dangerous complications [21-23]. However, a low prevalence of adequate control of BP has been documented in the USA, Spain, Canada, France and the UK [24-29], and this situation was reflected in the present study. Antihypertensive treatment could not prevent the complications of hypertension unless good control of BP was obtained, and combination therapy with ≥2 drugs was conducive to achieve optimal control of BP. This is consistent with the recommendations of JNC 7 [30]. More than 75% of patients preferred single-drug therapy, and only 22% of patients underwent multidrug combination therapy. Compound drugs were well received by Chinese farmers (44%). These compound drugs usually contain traditional Chinese medicines and Western medicines at a low dose (e.g., hydrochlorothiazide, hydralazine, reserpine). They are called "traditional compound drugs" (TCDs), have been used to reduce BP in China since the 1950s, and are widely accepted by Chinese people (especially farmers) due to their low cost and convenience. We revealed TCDs to be beneficial to good control of BP but their effects for lowering BP and protecting target organs (or their side effects) have not been confirmed by large clinical trials. The efficacy and tolerability of some compound antihypertensive drugs (e.g., losartan/hydrochlorothiazide, amlodipine/valsartan, irbesartan/hydrochlorothiazide, perindopril/indapamide) have been confirmed by evidence-based studies [31-34]. These compound products are low fixed-dose combinations of two antihypertensive drugs and are called "modern compound drugs" (MCDs). The cost of MCDs interferes with their application in Chinese rural areas despite their high efficacy and excellent tolerability. The monthly treatment cost of each MCD described above is >200 Yuan RMB. According to the maximum acceptable treatment cost of subjects in the present study, only 5.5% could bear the cost; >94% could not afford it.

Our survey suggested that some multidrug combination therapy protocols, such as combination therapy with nitrendipine and captopril (or enalapril), nitrendipine and metoprolol, or hydrochlorothiazide and captopril, were effective and sufficiently inexpensive to be afforded by most rural patients. However, multidrug therapy was considered to be expensive and inconvenient, and seldom accepted by most patients. If these inexpensive and efficacious drugs were made into compound products (e.g., nitrendipine + captopril, or nitrendipine + metoprolol) they could be readily accepted by most Chinese farmers. These compound drugs could improve the control of hypertension, and should be tried by Chinese health authorities.

This study had limitations. First, we did not exclude white-coat hypertension and secondary hypertension from hypertensive subjects. Second, among hypertensive subjects, adverse outcomes were probably missed due to incomplete tests. For the sake of excluding white-coat hypertension or secondary hypertension, some medical checks (e.g., ambulatory blood pressure monitoring, aortic MRI, adrenal CT) should be undertaken. Similarly, more target-organ damage could be detected if echocardiography, CT or MRI of the head, or the microalbumin urine test was carried out. However, this was an epidemiological study involving thousands of people. It is unrealistic to undertake such expensive investigations. Finally, organ damage (heart, brain, kidney) could have resulted from other illnesses (e.g., diabetes). However, it is quite common for hypertensive patients to also have diabetes. Among 1,340 hypertensive subjects detected, 128 subjects were urine glucose-positive and/or had a history of diabetes. The prevalence of diabetes among hypertensive subjects was 9.6%. Hypertension and diabetes interact, coordinate and contribute to target-organ damage. The individuals with diabetes could not be eliminated from hypertensive subjects in case the rest of the subjects were not representative of the total hypertensive population.

## Conclusions

Early intervention, such as health education and regular follow-up in clinic, can decrease the complications of hypertension. However, the cost of early intervention is ignored by the NRCMS. The present study showed poorer management of hypertension in rural women, and showed that the NRCMS contributes little to hypertension management. We suggest that the NRCMS should concentrate on early intervention of hypertension and to focus on women. To increase hypertension control in rural areas in China, compound products containing effective and inexpensive drugs (and not multidrug therapy) are needed.

## Competing interests

The authors declare that they have no competing interests.

## Authors' contributions

ZM conceived of the study, and participated in its design and coordination, and drafted the manuscript. MY participated in the design of the study and helped to draft the manuscript. YY designed questionnaire and took charge of the household survey. LY carried out the household survey and performed electrocardiography. DC participated in the household survey and conducted serum creatinine determination. XJ participated in the household survey and performed the statistical analysis. ZL participated in the household survey and helped to perform the statistical analysis. LF participated in the household survey and carried out the urine test. All authors read and approved the final manuscript.

## Pre-publication history

The pre-publication history for this paper can be accessed here:

http://www.biomedcentral.com/1471-2458/11/301/prepub
